# Evaluating the Injection of Platelet-Rich Plasma on Second Lower Molars Protraction: A Randomized Controlled Clinical Trial

**DOI:** 10.7759/cureus.57801

**Published:** 2024-04-08

**Authors:** Amer Khatib, Feras Baba

**Affiliations:** 1 Department of Orthodontics, Faculty of Dentistry, University of Aleppo, Aleppo, SYR

**Keywords:** power arm, miniscrews, growth factors, prp injections, mandibular molar mesialization, molar protraction

## Abstract

Background

The use of platelet-rich plasma (PRP) in dentistry was entered several decades ago, yet its clinical use in orthodontics still requires further investigation. The aim of this study was to evaluate the possible effects of local injection of PRP on the rate and type of mandibular second molar protraction movement compared with the comparator (no PRP) group.

Material and methods

Eighteen patients aged between 17 and 25 years were randomly allocated in a split-mouth study design to receive PRP injections on one side immediately before the start of molar protraction (PRP group), while the other side received only saline solution (comparator group). Eligibility criteria included bilaterally extracted mandibular first molars cases and indicated mandibular second molars protraction. The primary outcome of the study consisted of measuring the rate of molar protraction from the beginning of protraction (T0) to the end of the seventh month (TF), using a digital gauge. The secondary outcome included measuring the type of second molar protraction movement between T0 and TF by lateral cephalometric images. Randomization of the intervention side was performed by picking out opaque sealed envelopes. The blinding of the principal investigator was impossible but blinding of the patient was achieved by injection of saline. Analyses were done using paired samples T-test to compare the changes in all variables between T0 and TF. The level of significance was taken at a P-value < 0.05.

Results

No significant difference was detected between the PRP and comparator groups in the rate of second lower molar protraction during seven months (0.56±0.07 mm per month in the comparator group, whereas, was 0.6±0.11 mm per month in the PRP group). Molar protraction parameters in both groups showed second lower molars moving by controlled tipping closer to bodily movement (Root Movement:Crown Movement≈0.8).

Conclusions

Platelet-rich plasma (PRP) is ineffective in accelerating the rate of orthodontic tooth movement (OTM) during molar protraction, and it has no effect on the type of tooth movement. In addition, the mechanics that we used (6 mm power arm in combination with miniscrews) are effective in mandibular second molar protraction by controlled tipping closer to bodily movement.

## Introduction

The lower molars are the teeth most likely to be lost in adults, especially the lower first molars, as a result of caries and periodontal diseases [1]. Loss of lower permanent molars results in undesirable consequences such as mesial tipping, rotation of adjacent teeth, and supra-eruption of opposing tooth into the extraction space [2]. Treatment options for this condition involve restoration with a fixed partial denture or a dental implant. Orthodontic space closure of a remodeled edentulous space by second molar protraction into missing first molars is a viable treatment option, especially for younger patients, who will undergo orthodontic treatment for other malocclusion issues [3].

Orthodontic temporary anchorage devices (TADs) can provide skeletal anchorage for mandibular molar protraction, avoiding the problems often encountered with the use of dental anchorage such as reciprocal retraction of the incisors or shifting of the dental midline [4]. Even so, Mandibular molar protraction has been reported as a challenging procedure because of the molar’s large root surface area and density of mandibular bone. This makes it difficult to maintain root parallelism during mesial movement and prolongs treatment time [5].

Today, it is still very challenging to reduce the duration of orthodontic treatments, because of risks of caries, gingival recession, and root resorption. Various approaches have been attempted to accelerate the rate of orthodontic tooth movement (OTM). One of the recently used local agents to accelerate the rate of OTM is platelet-rich plasma (PRP) [6]. It has been hypothesized that PRP can simulate the effects induced by surgical therapy without having the disadvantage of invasiveness or aggressiveness seen in different surgical approaches [7].

PRP is defined as an autologous concentration of platelets in a small volume of plasma. It is considered to be a rich source of autologous growth factors (GFs) [8]. The action of PRP is derived by the degranulation of cellular alfa-granules consisting of GFs and cytokines. PRP was shown to increase the proliferation, viability, and migration of mesenchymal stem cells, and promote angiogenesis, osteogenesis, and bone regeneration [9]. The multitude of GFs within the PRP has the potential to stimulate both osteoblastic and osteoclastic activities [10]. The aim of this study was to evaluate the possible effects of local injection of PRP on the rate and type of movement of second molar protraction compared with the comparator group (split-mouth study).

## Materials and methods

Study design and settings

The current study was a split-mouth randomized controlled clinical trial that involved the evaluation of the effects of injection of PRP on mandibular second molar protraction. This study was carried out at the Dental College and Hospital, University of Aleppo, Syrian Arab Republic between June 2020 and February 2023.

The clinical procedure began after obtaining formal approval from the Local Ethics Committee at the University of Aleppo Dental College, Syria (Ref no. UADS-2401-08032020/SRC-1036). The study is also registered on ISRCTN registry.com (ISRCTN47495953).

Sample size

The sample size was estimated using Minitab® 17 software (Minitab Inc., State College, PA, USA), with the following assumptions: the one-sample t-test was applied, the statistical power of 99%, and a significance level of 0.05 was assumed. The rate of second molar protraction per month was collected from Al-Areqi`s study data [11]. The software showed that the required sample size was 15 patients. To compensate for sample attrition, the number was increased to 18.

Patient recruitment and eligibility criteria

Recruitments were selected by examining patients who had visited the Department of Orthodontics at the University of Aleppo Dental College and who sought orthodontic treatment. 18 participants were identified to be eligible for the study. Because three patients were excluded due to miniscrews failure, 15 of them completed the study and were analyzed. The recruited patients were three males and twelve females. The included patients were randomly allocated in a split-mouth study design to receive PRP injections on one side (PRP group), while the contralateral side received only saline solution (comparator group). The split-mouth design reduces biological variables between the intervention side and the contralateral comparator side and therefore requires a lower sample size [12]. The methods that were used remained unchanged throughout the study.

The inclusion criteria were patients aged between 17 and 25 years, who have all lower permanent teeth present, including intact third molar, but the bilateral first permanent lower molar was extracted and indicated second molar protraction, in which extractions were done within at least one year to ensure complete extraction socket cortication and at most three years to avoid severely inclined second lower molars, or severely reduced alveolar bone height and width.

The exclusion criteria were patients who have undergone previous orthodontic treatment or have poor oral hygiene, and systemic disease especially those with coagulation problems or who are being treated with anticoagulants and NSAIDs, as well as patients with unilateral chewing or extra-functional habits.

The eligible patients were informed about the procedures of the study. Patients' agreement was obtained by signing papers before recruitment and the patients were informed that the data will be used and published in this study.

Randomization of the intervention side (split-mouth design) and blinding

Patients were given numbers according to the historical sequence of their receipt. Then the side of the PRP application (right or left) was determined by randomly drawing an opaque sealed envelope from a container for the first patient, who was on the right side, so the left side was a comparator group. The side of the application is reversed to the next patient in the numerical sequence.

Orthodontic treatment in both PRP and comparator groups

In all patients, fixed metal orthodontic brackets (IOS, Boston, USA) with MBT prescription 0.022 were bonded. Initial alignment was achieved with a series of nickel-titanium archwires, increasing in size to 0.019×0.025 stainless steel. Before the commencement of the protraction, the archwire was left in situ for at least one month to become passive. Occlusal interferences were checked regularly and if present.

Titanium miniscrews (diameter 1.6 mm; length 8 mm; Genesis; Dentos, Daegu, India) was screwed through the bone on the labial surface of the mandibular alveolar ridge between the roots of mandibular canine and first premolar in all the patients. The choice of miniscrew depended upon the space availability between the roots after apical radiographs were taken.

**Figure 1:**
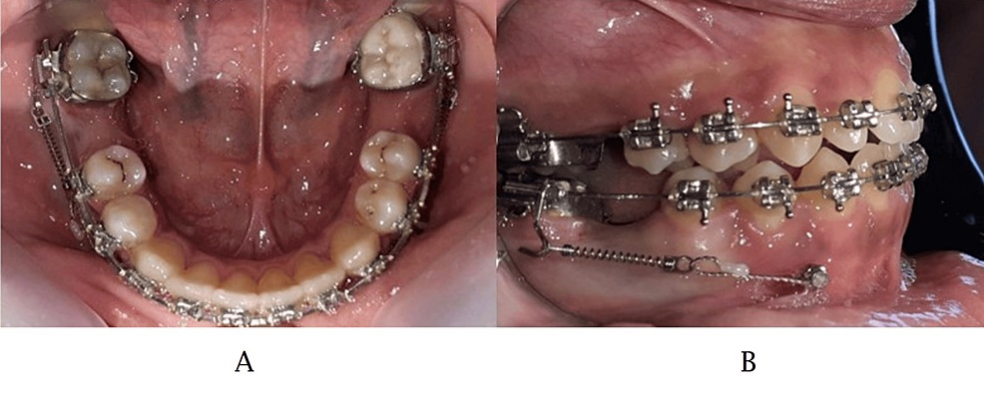
The mechanics that we used for mandibular molar protraction. (A) Horizontal view, (B) lateral view

In order to bring the force vector closer to the center of resistance in the system, a handmade power arm 0.021 × 0.025-inch SS, was inserted into the axillary tube of the second lower molar band. A closed nickel-titanium coil spring was activated to deliver 200 g of force and attached from the mandibular second molar power arm to the head of the miniscrew (Figures 1A, 1B).

PRP preparation and injection for the PRP group

**Figure 2:**
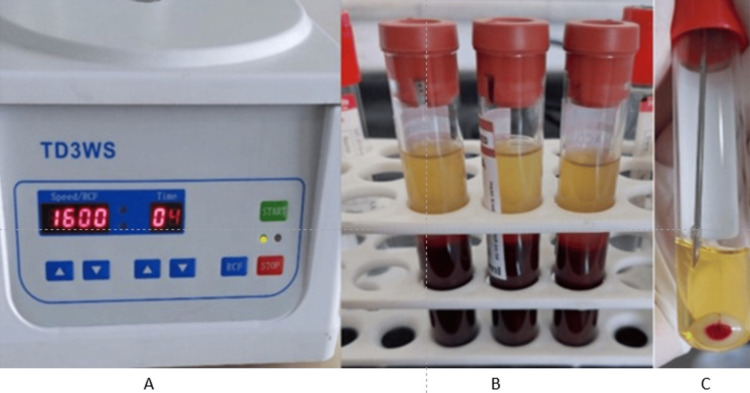
PRP preparation (A) The blood was centrifuged by the double-spin technique. (B) Separation of blood components into 3 different layers after first centrifugation. (C) PRP drawing after second centrifugation.

20 ml of blood was directly collected from the patient by butterfly scalp vein to sterile tubes with anticoagulant citrate dextrose solution (ACD-A) as an anticoagulant. One milliliter of the blood sample was set apart to determine the concentration of platelets. PRP was prepared by the double-spin technique described by Marx and Garg [13] with modifications; initially, the blood was centrifuged at 1,600 rpm for four minutes to separate the plasma containing the platelets from the red cells. The plasma was drawn off the top and centrifuged for an additional six minutes at 3,500 rpm to get PRP (Figures 2A-2C).

**Figure 3:**
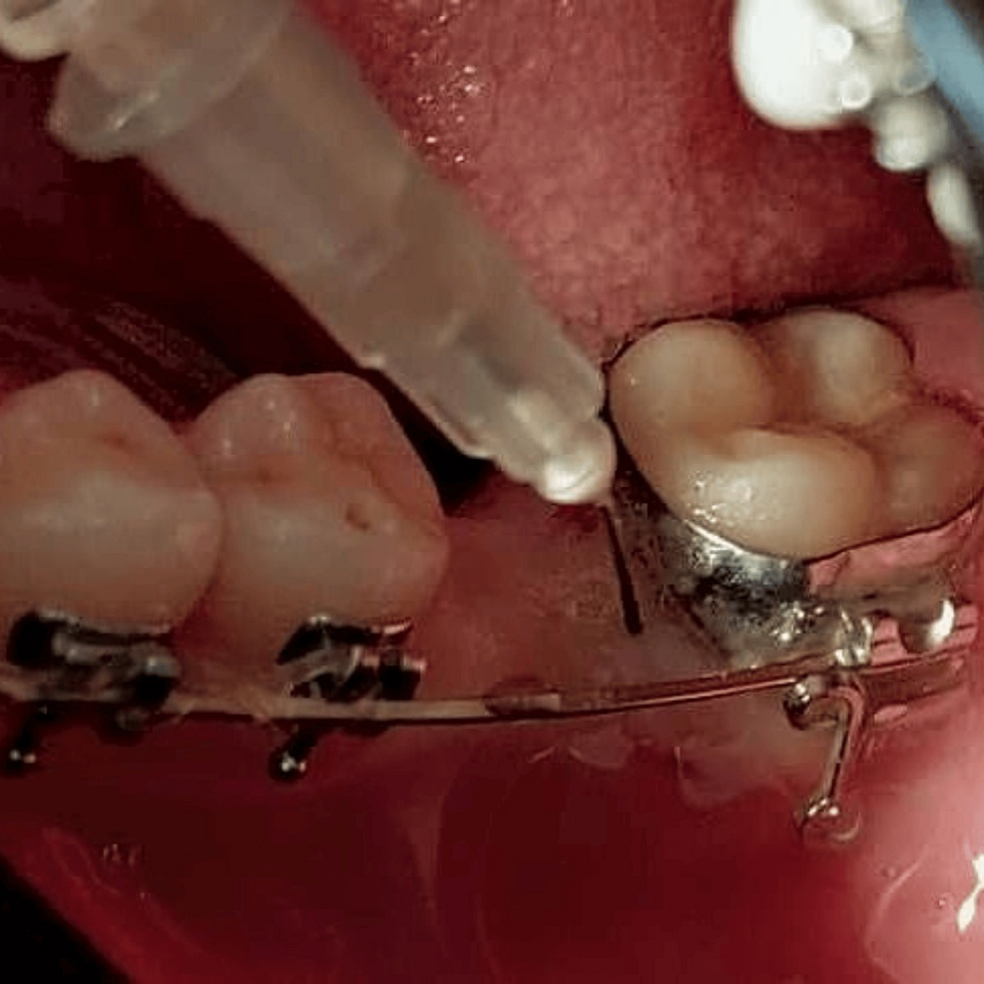
PRP was injected into the areas of the mandibular second molar

The intervention site was anesthetized, and 0.5 mL of PRP was slowly injected submucosally into the mesiobuccal, mesiolingual, mesiomedian, and bifurcation areas of the mandibular second molar (Figure 3). In contrast, the other comparator side was injected with only by saline solution. Paracetamol was prescribed for the patient to control pain and ensure confirming not to use ibuprofen or another NSAIDS. The injection was applied once time throughout the study.

Outcome assessment

The amount and rate of molar protraction were evaluated from the difference in the mandibular second molar position at the beginning of protraction and the end of the seventh month, using a digital gauge (Anyi model 110-011, Guangxi, China) to measure the mesial-distal length of edentulous space between contact points of the distal second premolar and mesial second molar. The measurement was repeated more than once by another researcher.

Cephalometric variables

**Figure 4:**
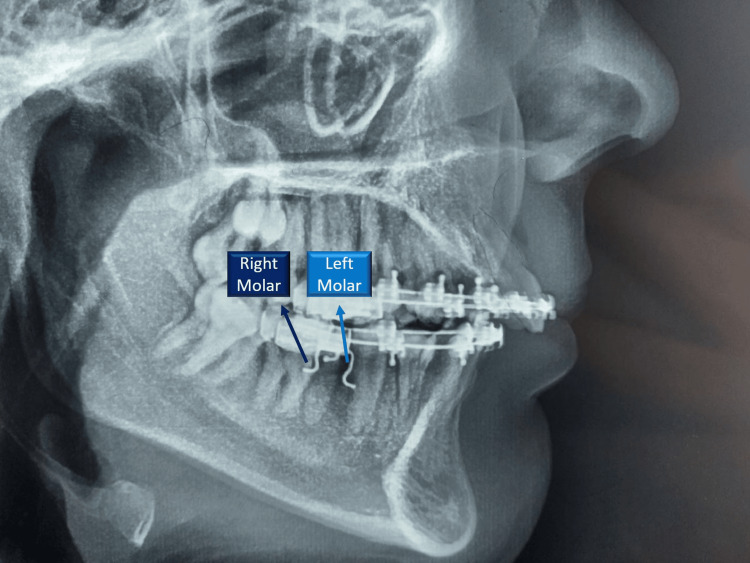
Differential wires inserted into auxiliary tube of molar band to determine the right and left mandibular second molar

The skeletal, dentoalveolar, and soft tissue changes were evaluated using PaX i3D Green (VATECH Corporation, Ltd., Hwaseong, Korea) for lateral cephalometric acquisition with the same settings. Each patient took lateral cephalometric images before molar protraction (T0) and after seven months of molar protraction (TF). We manually traced the right and left mandibular second molar after adding differential wires to determine both sides (Figure 4).

**Figure 5:**
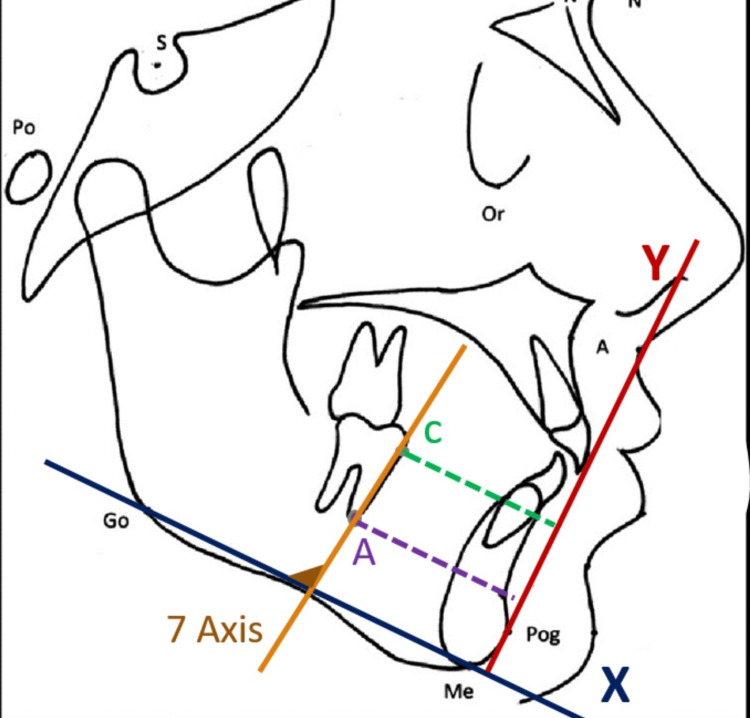
Cephalometric dental landmarks, planes, and sagittal and angular measurements X-plane: which is mandibular plane (Go-Me) Y-plane: perpendicular line was erected from X through Pog C: The most mesial point of the mandibular second molars A: The most inferior point on the mesial root of the mandibular second molars 7Axis/X: The angle between the mandibular second molar axis CA and mandibular plane X

The following points were used in this study (Figure 5): Menton, Gonion, Pogonion, C (the most mesial point of the mandibular second molars), A (the most inferior point on the mesial root of the mandibular second molars) [14]. The planes drawn were the X plane which is a mandibular plane (Go-Me), and the Y plane perpendicular line was erected from X through Pog.

The following millimetric and angular measurements were made for the right and left molars (PRP group and comparator group) before molar protraction T0 and after seven months of molar protraction (TF) (Figure 5):

C (Crown)/Y: Distance between C point and perpendicular line Y.

A (Root)/Y: Distance between A point and perpendicular line Y.

So, Crown Movement (CM) is the difference in Crown/Y measurement between T0 and TF, and Root Movement (RM) is the difference in Root/Y measurement between T0 and TF.

To evaluate the anteroposterior type of OTM of the mandibular second molar on the lateral cephalogram was determined by the ratio (RM: CM). Also, the angle (7Axis/X) between the second mandibular second molar axis CA (7Axis) and mandibular plane X was measured for the right and left molars (PRP group and comparator group) in T0 and TF. All cephalometric measurements were done manually.

Assessment of the reproducibility of the measurements

The Reliability of the Measurements

The reliability of the measurements was studied using Cronbach's Alpha coefficient. The reliability value in all measurements ranged between 0.89 and 0.997. Therefore, the reliability of the measurements can be accepted.

The Error of the Method

The error of the method was evaluated using Dahlberg formulas on 10 randomly selected cephalograms. These radiographs were remeasured by the other principal researcher, and it was ranged from 0.2 to 0.28 which considered low. Whereas intraclass correlation coefficients (ICCs) ranged from 0.988 to 1. No systematic error was detected using the paired t-test.

Statistical analysis

Shapiro-Wilk test demonstrated normal distribution for all variables (P>0.05 for all of them). The resulting data were statistically analyzed using SPSS software version 25.0; IBM Corp., Armonk, NY). Descriptive statistics including means and standard deviations were calculated for every variable incorporated in this study. Analyses were done using paired samples t-test to compare the changes in all variables between T0 and TF. The level of significance was taken at P-value < 0.05.

## Results

**Figure 6:**
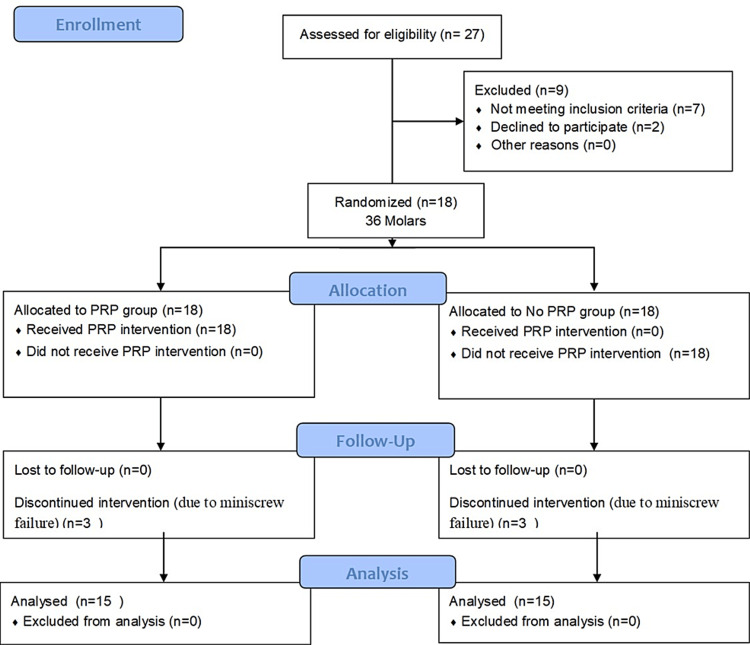
Consolidated standards of reporting trial (CONSORT) participant's flow diagram.

The clinical trial's flow chart which outlines the investigation process, is shown in Figure 6. A total of 27 patients were assessed for eligibility, seven patients did not meet the inclusion criteria and two patients declined to participate. The remaining 18 patients were allocated into two groups according to the treatment received (n=9). Fifteen patients completed the trial and were available for final analysis Because three patients were excluded due to miniscrew failure.

Rate of molar protraction

The mean amount of mandibular molars protraction from T0 to TF (during seven months) in the comparator group was 3.92±0.53 mm, whereas it was 4.22±0.81 in the PRP group Table 1. Thus, the rate of the molars protraction was 0.56±0.07 mm per month in the comparator group, whereas was 0.6±0.11 mm per month in the PRP group. The mean molar protraction showed a nonsignificant difference between the two groups (P>0.05) (Table 1).

**Table 1:** Descriptive statistics, mean differences, and P-values for amount and rate of molars protraction in PRP and comparator groups from T0 to TF SD: Standard deviation, Sig: Significant difference, NS: Not-significant (P>0.05)

Variable	Groups	Mean ±SD	Mean Differences	P -value	Sig
Amount of molars protraction	Comparator Group	3.92±0.53 mm	-0.30	0.216	NS
	PRP Group	4.22±0.81 mm			
Rate of the protraction per month	Comparator Group	0.56±0.07	-0.04	0.230	NS
	PRP Group	0.60±0.11			

The type of movement of molar protraction

The type of mesiodistal movement of molar protraction was measured by a ratio RM: CM, and it was controlled tipping close to bodily movement in both comparator and PRP groups. The ratio RM: CM was 0.78±0.89 in the Comparator Group, whereas in the PRP group was 0.79±0.80, An intergroup comparison for protraction movement type showed statistically insignificant differences between both groups (Table 2).

**Table 2:** Descriptive statistics, mean differences and P-values for the mesiodistal type of molars protraction by measuring the mean RM:CM ratio in both groups from T0 to TF Sig: Significant differences, RM: Root Movement, CM: Crown Movement, NS: Not-significant (P>0.05)

Variable	Groups	Mean ±SD	Mean Differences ±SD	P-value	Sig
Crown Movement (CM)	Comparator Group	3.95±1.07	-0.43±1.33	0.22	NS
	PRP Group	4.32±1.37			
Root Movement (RM)	Comparator Group	3.08±0.93	-0.3±1.5	0.44	NS
	PRP Group	3.40±1.36			
RM:CM	Comparator Group	0.78±0.89	-0.01±0.6	0.97	NS
	PRP Group	0.79±0.80			

Molar angulation

There was an insignificant difference in mesiodistal angulation of the mandibular second molars of 2.76° in the comparator group, whereas 2.56° in the PRP group between T0 and TF. An intergroup comparison for molar angulation showed statistically insignificant differences between both groups (Table 3).

**Table 3:** Descriptive statistics, mean differences and P-values for mesiodistal angulation of the mandibular second molars 7Axis: Mandibular second molar axis, MP: Mandibular plane (Go-Me), NS: Not-significant (P>0.05)

			Means ±SD		MD	P-value	Sig
7Axis/MP	Comparator Group	T0	76.30	-2.76±7.02	0.2	0.91	NS
		TF	79.06				
	PRP Group	T0	75.63	-2.56±4.93			
		TF	78.20				

## Discussion

Effect of PRP on OTM

Although PRP has been used extensively in various fields of dentistry, showing vast advantages and applications [15,16], the efficacy of PRP on OTM acceleration remains controversial based on the current limited evidence. Therefore, more well-designed randomized controlled trials involving humans are called for to obtain more clinically significant conclusions.

This is the first clinical trial to evaluate the effects of injection of PRP on the rate and type of mandibular second molar protraction movement over a seven-month period. Previous literature revealed that the techniques for preparing PRP have varied, all with the intention of standardizing the PRP preparation procedure [17].

In the current study, we found that the number of blood platelets produced after the second stage of the centrifugation protocol is approximately five times the number of platelets present in the blood sample of the same patient which is around one million per milliliter. This agrees with Marx's definition of PRP as a working functional PRP [8]. Whereas, we disagreed with Liou's opinion, who considered that the platelet count in the plasma sample should be 9.5 to 12.5 times greater than its count in the blood sample, without providing a clear clinical study [7].

We used ACD-A (Anticoagulant Citrate Dextrose) Solution because it Contributes to supporting the vitality of platelets, and could not damage the platelet membrane [8,17]. We injected saline solution because the injection process itself is a confounder with a tissue injury effect that could impact the outcome.

We did not perform any further reinjection because PRP remains active for approximately six months, and the duration of our study was seven months post-injection [7,18]. Also, we want to investigate the effect of a single PRP injection on molar protraction.

In this study, the overall rate of OTM was slightly greater in patients who received PRP compared to the patients who did not, but it was not statistically significant (P > 0.05). The findings of the current study were in agreement with Chandak et al. who suggested that PRP is ineffective in accelerating the rate of OTM [19]. Also, Akbulut et al. reported that the application of PRP was not beneficial as an adjunct to orthodontic treatment because PRP does not affect osteoblast and osteoclast cell counts, and ALP, TRAP, and TGF-b expressions [20]. Sufarnap et al. found that tooth movement was similar with or without PRP injection on days 6, 9, 12, and 24 after rubber separator placement [21]. On the other hand, this finding contradicts Güleç et al. who reported that, on day 21, the amount of tooth movement induced by high concentrations of PRP was 1.7 times more than that on the comparator side [22]. Rashid et al. also reported a higher percentage of mean changes in tooth movement than the comparator group, with a percentage change ratio of 2.13:1 [6]. El-Timamy et al. found that despite the statistically significant increase in the rate of canine retraction during the early stages of tooth movement concomitant with PRP injections, PRP did not exhibit long-term acceleration effects [23]. Our study findings may be related to different factors as in the following.

Inadequate Concentration of PRP

In our study, we got PRP in a concentration fivefold higher than baseline levels, where higher concentration PRP could accelerate OTM by decreasing the alveolar bone density on paradental tissues by enhancing osteoclastic activity in a transient way [22].

Structure of Mandibular Bone

Most scientific studies have focused on examining OTM acceleration on the upper jaw. There is a general agreement that the rate of OTM in the lower jaw tends to be slower due to differences in remodeling rate and bone density of the alveolar bone facing the movement. Where the cortical bone thickness in the vestibular region and the lingual region of the second lower molars reached 1319 and 1281 Hounsfield units (HU), respectively [24]. This makes it challenging for GFs released from blood platelets to penetrate and affect the acceleration of OTM.

Type of mandibular second molar protraction movement

The major concern regarding molar protraction is the need for an adequate anchorage unit to avoid anterior anchorage loss [25]. So, in this study, mini-implants were applied to provide absolute anchorage during second molar protraction. A closed NiTi coil spring was used to achieve molar protraction since it provides a constant and more predictable amount of force [26].

To achieve the maximum amount of bodily movement, mandibular second molar protraction was carried out using a rigid rectangular SS archwire and a rigid power arm to apply traction at the molar furcation level closer to the center of resistance. If forces had been applied at the coronal level, molars would have tipped mesially during protraction, which causes archwire binding and consequently mesial transfer of the entire dental arch (molar extrusion and incisor intrusion) [27].

In this study, we found that the type of tooth movement was controlled tipping close to bodily movement in both comparator and PRP groups with the ratio RM: CM was 0.78 and 0.79 respectively. The little mesial tipping of mandibular second molars may still occur due to the playing angle between the archwire and the molar tube.

Previous studies suggested that molar protraction often requires a greater amount of RM than CM to achieve bodily movement with molar uprighting [5,14,28,29] in disagreement with our study. However, the timing for collecting records and evaluating results in our study was after completing alignment, leveling, and correcting any tilting of the teeth. In other words, T0 was at the start of effective protraction for the second lower molars, not at the beginning of orthodontic treatment as in previous studies in which T0 was before orthodontic treatment. Nevertheless, those studies did not exclude severe tipping molars.

This was done to conduct a precise assessment of the effectiveness of the mechanics used in molar protraction and the impact of plasma injection on the OTM process. Also, we did not utilize any uprighting molar springs or class II elastics during molar protraction. Additionally, no movements were performed on the upper jaw teeth throughout the study period. Thus, isolating any impact of other confounding factors on the effectiveness of our study mechanics.

Furthermore, limitations of the current study include the greater female-to-male ratio and the inability to blind the principal investigator. Additionally, the degree of molar rotation has not been measured in this study, and the follow-up period did not continue until complete molar protraction.

## Conclusions

Within the limitations of the study and by evaluating the efficiency of PRP on second lower molars protraction in comparison with no PRP group and on the basis of the study results, the following conclusions can be drawn: PRP showed a minimally but not statistically significant increase in molar protraction rate compared with comparator group. The mechanics that we used in mandibular second molar protraction (6 mm power arm, miniscrews, 19×25 SS archwire) managed to achieve approximately bodily movement (RM:CM≈0.8), thus avoiding negative side effects on the dental arch.
